# State of Diseases in the Bristol Dispensary

**Published:** 1801-08

**Authors:** W. D. Rolfe

**Affiliations:** Bristol


### *To the Editors of the Medical and Physical Journal.*

GENTLEMEN,

SOME more striking proofs of the efficacy of yeast in typhus and putrid fevers have lately occurred in my practice; if thought worthy a place in your Journal, you will oblige me by inserting them.

Having under my care a young man ill with Typhus fever, belonging to a large family, I soon perceived many of them falling victims to it. Being obliged to visit the house every day on the young man's account, and finding two more taken very ill, I recommended them the use of yeast, as I was prevented assisting them otherwise from their not being able to procure Dispensary notes. In the course of two or three days, the father, mother, and another child fell ill; and as they had only one room for all six to lie in, two of them were carried to the Infirmary, and I procured Dispensary notes for the other three. Finding the two who were removed to the Infirmary had been so much relieved by the yeast before they were taken away, I was induced to try it with the others, who were delirious nearly the whole of the time. I persevered in the use of the yeast, without any thing else, until after the crisis was over, when I gave them an infusion of bitters for a few days, and they all perfectly recovered. I am, &c.

*Bristol*, 10, 1801. W. D. ROLFE.

*State of the Patients of the Bristol Dispensary, from the 1st of June to the 1st of July,* 1801.

| | | | |
|---|---|---|---|
| Typhus Fever | 43 | Puerperal Fever | 1 |
| Rheumatism | 5 | Asthenia | 7 |
| Tussis | 1 | Variola | 8 |
| Cardialgia | 2 | Febris Simplex | 9 |
| Gastrodynia | 2 | Inflamed Breasts | 2 |
| Cephalalgia | 1 | Ascarides | 1 |
| Anasarca | 3 | Menorrhagia | 3 |
| Ascites | 2 | Pluretic | 3 |
| Diarrhœa | 3 | Hydrops Ovaria | 1 |
| Peripneumonia | 3 | Gravel | 1 |
| Podagra | 1 | Cynanche Maligna | 3 |
| Consumption | 8 | Mania | 1 |
| Ulcerated Sore Throat | 1 | | |

